# Family Reflections on a Lifecourse Journey after Neonatal Intensive Care: Neurodiversity, Enablement and Hope

**DOI:** 10.3390/children11020165

**Published:** 2024-01-26

**Authors:** Michael E. Msall

**Affiliations:** Section of Developmental Pediatrics, JP Kennedy Research Center on Intellectual and Neurodevelopmental Disabilities, Comer Children’s Hospital, University of Chicago Medicine, 950 East 61st Street Suite 207, Chicago, IL 60637, USA; mmsall@bsd.uchicago.edu

**Keywords:** life course health development, preterm birth, counseling about developmental disability, family support, high-risk infant follow-up, neurodiversity, enablement, functioning, participation, adult outcomes

## Abstract

In 1969, my sister Christianne was born late preterm with a genetic disorder and given a very pessimistic prognosis. I will describe, from a family perspective, some lifecourse lessons about neurodiversity using the World Health Organization International Classification Model of Functioning (WHO-ICF). This model emphasizes that, in communicating about the complexity of outcomes of disability, attention must be paid to facilitators and barriers for optimizing health, functioning in daily life, and participation in the community. I will describe several developmental lifecourse lessons learned in negotiating fragmented systems of health, education, and community care. I will suggest ways to improve physician–parent communication, focusing on enablement to decrease a family’s sense of isolation and despair. I have benefitted from my parents’ archives, discussions with all my seven sisters (including Christianne), and discussions with my brother and sister-in law. They all have provided invaluable feedback from a family perspective during Christianne’s lived lifecourse journey with neurodiversity.

## 1. Introduction

In 1969, the day after I graduated from high school, my sister Christianne was born late preterm with a genetic disorder. This impacted her lifecourse trajectory, my personal experiences as her brother, and my ongoing professional identity. I became a pediatrician and then a specialist in neurodevelopmental medicine. My sister Camille, age 63, and I have become Christianne’s guardians since my father’s death in 2001. My sister Karin, age 68, and I have become her co-medical powers of attorney since my mother’s diagnosis of dementia in 2011. With Christianne’s participation and ongoing family dialogues, we have maintained her community support needs as her medical and neurobehavioral functioning have become more complex. In these matters, we continue to implement Christianne’s and my parents’ shared goals for optimizing health, social inclusion, and living with a community of peers and neurotypical housemates. As I reflect on celebrating her 54th birthday and my 40 years in academic medicine, I will describe some lessons I have learned. I will do this from a lived experience framework using the WHO-ICF Model [1] and paying explicit attention to the impact of neurodiversity after prematurity on families (including my siblings) over key lifecourse epochs. I will describe the uncertainties in the current systems of fragmented health, developmental, and community care. I will also suggest ways to improve physician–parent communication to decrease a family’s sense of isolation and despair with an emphasis on a lived lifecourse journey with neurodiversity. This family perspective includes my parents’ archives, discussions with all my seven sisters (including Christianne), and discussions with my brother and sister-in law.

## 2. Beginnings: Partnering with Families

The first lesson I learned occurred in the first 48 hours of life at the time of Christianne’s diagnoses. Christianne had been born 4 weeks preterm and demonstrated a loud cardiac murmur with an abnormal neurological exam. Our family physician made the following prognoses to my parents: “Your daughter has microcephaly, a loud atypical heart murmur and a probable genetic disorder.” It should be noted that, at this time, there was no echocardiography, and the detection of the murmur was felt to indicate severe congenital heart disease. The physician prognosticated that Christianne would experience severe mental retardation (now known as intellectual disability). Because of her heart disease, he prognosticated that she would not be healthy, struggle with growing, and experience frequent pneumonias. As there were few options for pediatric open-heart surgery before 2 years of age, he prognosticated that Christianne had a high risk of death. Even if she survived, he stated that Christianne would not learn, go to school, or participate in community activities. When my parents asked what might happen after hospital discharge, he stated that my parents should take Christianne home, love her, and not expect her to progress developmentally. My parents experienced grief and shock. I will return to their journey of coping later, but I will now describe several themes that require much more thought and reflection from neonatal professionals. These involve communicating difficult diagnoses, frameworks for understanding lived experiences with a spectrum of neurodevelopmental outcomes, and the importance of reviewing an individual’s whole-person status across the challenges of health and development over preschool, middle school, adolescent, and adult years. In all these themes, there is a common link—the need to take a lifecourse health development approach.

***Lesson 1: Be careful about prognostication when there is uncertainty.***

From the initial pessimism of Christianne’s prognoses in the Newborn Intensive Care Nursery, I have learned many lessons. As a brother and future health professional, I learned that health professionals must be extremely careful when communicating about the uncertainty of future prognoses [2,3,4]. This is critical when professionals have not cared for thousands of individuals longitudinally across epochs of childhood, adolescence, and adulthood [5,6]. This lack of prognostic information that informs counseling about the spectrum of neurodiversity holds for infants at the limits of viability, infants with congenital malformations requiring surgery, and infants with severe neonatal complications, including brain neuroimaging abnormalities. This is also especially important when there is a range of uncertainty especially with respect to different eras of medical and educational care [7,8,9]. Most importantly, parents and families require compassionate care, honesty about the accuracy of precise predictions, and explicit messages that medicine will be there as a resource for health, developmental, and family support. In this era of far-from-adequate medical homes and community support, health professionals must create shared decision making with practical updates across systems of care [10,11,12,13,14,15,16,17], Health professionals need also to recognize that past practices of communicating “the bad news about disability” in a few short counseling sessions before discharge is not sufficient [18,19,20]. They benefit from both simulated experiences and broader literature including lived experiences about disability and shared decision making [11,12,13,14,15,16,17]. Too often, health professionals fail to acknowledge that much past information about intellectual disability left families stigmatized and isolated, and often blamed parents for not accepting outdated information [21,22]. In this context, health professionals were unaware of discriminatory practices in health, education, and rehabilitation that were too often self-fulfilling prophecies and too often did not provide the emotional and physical presence of caring over time. [16,17,23,24,25]. Some guidelines for communicating uncertainty in settings of disability to families are highlighted in Table 1.

***Lesson 2: Beware of the limitations of current evidence about long term outcomes.***

What is often forgotten in both past and current practices of physicians communicating about developmental disabilities is the range of outcomes and limitations of what our clinical care and technology predict. Too often physicians do not acknowledge their ignorance about the range of health and developmental outcomes and some of the biases underlying the literature [27,28,29]. First, health professionals experience limited longitudinal care exposures during their hospital training. During this training, they appropriately learn to recognize and stabilize critical physiological decompensations. Although there is increased attention to the complexity of communicating uncertainty in settings of high risks for disability and mortality [13,14,15,30,31], information necessary to understand future outcomes requires ongoing contact with children and families after they leave the hospital. Too often are there no informed experiences to allow them to learn about what happens with children and families on the journey of neurodiversity during developmental epochs of infancy, early and middle childhood, adolescence, and adulthood [32,33]. This lack of longitudinal experiences leaves little time to wonder regarding the following questions: how does health change; what skills, despite developmental challenges, does the child learn; how are families doing when the child goes home; what do families find helpful when the model of caring is not curative?

***Lesson 3: Establish registries for child health, development, and family outcomes.***

In order to understand the trajectories of critically ill or medically complex patients, it is essential to establish information systems that include multicenter registries with longitudinal tracking systems for capturing updated physical, developmental, behavioral, and social health outcomes. These minimal essential datasets must also include the medical, developmental, and rehabilitation supports received and include sequential measures of childhood functioning during daily routines. This latter category of person-centered activities includes seeing, moving, manipulating, communicating, and learning developmental, social adaptive, and regulatory behaviors. This use of multicenter registries is best highlighted in the Australia Cerebral Palsy Register (ACPR) [34,35]. A key outcome of these informed biopsychosocial information systems is to understand, on a population level, how children and families are faring with respect to optimizing their health, developmental functioning, and behavior outcomes [36,37]. Equally as important is how families are doing in accessing community support and feeling supported for their well-being [25,27,38].

Embedded in these longitudinal child health, development, and family well-being outcome registries will be data from representative contemporary populations, indicating that prenatal and neonatal impairments alone do not accurately predict long-term learning, the degree of independence in daily functioning, and family well-being [14,15,16,17]. This is certainly true for my sister and many children with congenital heart disorder, sensorineural hearing loss, and risks of a spectrum of motor, communicative, cognitive, and social adaptive disorders. Critically important for physicians was their lack of training and experience with the consequences of language that communicated stigma, isolation, cluelessness about community resources, and hopelessness [26,39,40].

## 3. Leaving the Hospital without a Roadmap for Community Support

My parents built on past experiences where physicians had predicted grim outcomes that did not occur. My father himself had experienced a critical illness with severe burns at age 8, and physicians considered bilateral above-the-elbow amputations when there were no antibiotics for wound care. My mother, at age 6, had spent several months in a rheumatic fever chronic disease hospital. Both my parents had lived full lives after being given poor prognoses. They both were healthy after these events, graduated from high school, and became teachers after college. Because of their personal experiences, religious beliefs, and professional activities, they believed, after a period of mourning, that they could help Christianne learn. She would be part of family daily routines and experiences, even if there was an uncertain prognosis.

My oldest sister, Anita, then 17 years old, and I, then 18 years old, became designated godparents at Christianne’s baptism. This resulted in two important family lessons. First, Christianne would not be isolated and would be a part of our community by becoming a member of our parish. Second, my parents shared with all of us that we would all work together to help Christianne learn. My parents, brother, and sisters all believed that this was how we would help any new family member—but especially Christianne.

What did my parents and siblings do? First, we decided that we would treat Christianne as capable of learning and involve her in all family routines. As my parents were teachers, they also decided to optimize the management of her medical conditions and participate in the best developmental/habilitative community programs. Her medical management involved accessing numerous otologic surgeries for her conductive hearing loss, the early use of hearing aids for her sensorineural hearing loss and celebrating her improved cardiac prognoses when her septal defect spontaneously closed. In seeking out best developmental treatments, my parents discovered that they could optimize Christianne’s learning through activity-based early interventions at home through a parent support group program [41,42].

## 4. Early Childhood and Developmental Accomplishments

At the age of 9 months, Christianne rolled both ways and could sit with supports. She could babble and laugh. At 12 months of age, she could sit hands-free and manipulate objects. She imitated basic gestures and liked to play peek-a-boo. At 15 months of age, she could crawl, finger feed, and say mama specifically. At 18 months of age, she could walk independently. At 24 months of age, she used three words, including more, me, and no. At 30 months of age, she enjoyed board books and Sesame Street songs. She had a vocabulary of 20 words and talked in phrases. At 3 years of age, she talked in sentences and enjoyed riding a big wheel bike. These positive neuromotor and prelinguistic experiences convinced my parents, as well as all the family members, that Christianne could and would continue to learn. The message from these accomplishments can be emphasized as

***Lesson 4: Celebrate developmental achievements with families.***

A major gap for physicians involves ways to touch base and support families during initial health and learning trajectories after hospital discharge and during early childhood. This needs to happen for several reasons. First, when families initially listen to unexpected news about complex health and developmental challenges, they experience not only the loss of their hopes and dreams but also the uncertainty of the road ahead.

Physicians require a more structured approach to teaching counseling that incorporates adult learning theory, emphasizes multidisciplinary teams on site with a simulation that links counseling to clinicians’ daily practice, and includes critical reflection, debriefing, and community outcomes [14]. Multiple educational strategies that train clinicians in advanced communication and decision making can offer promising experiences for optimizing counseling and shared decision making, not only for families facing prematurity but also for families with genetic disorders or neonatal encephalopathy [13,14,15,30,31].

Sullivan et al. have emphasized the need for reassurance and compassionate communication while parents travel along a dynamic decision-making journey that is ever-changing [14,15]. They highlight related themes of balancing positivity and negativity, tailoring the amount of information so as to avoid assumptions, inferences surrounding language, and values that can cloud the counseling process. She emphasizes the use of visuals to better anticipate and prepare for organ-specific challenges. She also highlights how team synergism is essential for maintaining trust and communicating consistently among medical, nursing, and social work professionals.

Feudtner has also emphasized that the major task is simple: be straightforward, clear, balanced, and compassionate [16,17] He emphasizes staying focused on helping parents as they search for a way to be the best parents they can be, on their own terms, for their child. He promotes, through narrative medicine, the critical importance of being fully present in the midst of strong emotions and stressors. This requires a determined reflective manner across challenging circumstances. He highlights that, even amid the tumult of some of the worst that life puts in front of us, some of the best that life offers also blooms [16]. This is also described in several other narratives among an extensive literature in medicine and disability studies [32,33,40,43].

When experiencing the journey of lived experiences with developmental challenges, families need messages of hope and antidotes for their worst fears. This primal fear is often not explicitly revealed by many parents of children with complex disorders and includes dread that the child will show no progress, have ongoing pain that cannot be comforted, and have a high probability of crisis emergency department encounters and frequent hospital readmissions. In contrast to these fears, Christianne’s ongoing health and developmental trajectories after neonatal discharge indicated a very different picture of real-life experiences [25,27]. My parents, siblings, and I learned throughout Christianne’s first and second years of life that she could accomplish developmental skills and did not require any emergency visits or overnight hospitalizations. We also learned that, although my sister experienced developmental challenges, she became self-mobile, learned language to communicate basic needs, and benefitted from play and learning through manipulating blocks, solving puzzles, having her dolls and stuffed animals participate in playing kitchen, coloring, and playground activities. Despite fears of severe limitations because of her developmental disability, Christianne made progress in her learning and functioning. It was important, during these health encounters, that physicians looked past their initial prognoses and began to understand that our family’s journey included moments of hope and joy [43].

## 5. Preschool and Complex Health Care Coordination for Optimizing Health

My sister required frequent middle ear surgeries and sedated ABRs under anesthesia, as well as the removal of her tonsils and adenoids. Her otolaryngology needs were managed by outpatient surgery. Since my sister’s responses to conversations improved after these surgeries, we learned that health care was more than critical illness. Overall, before age 21, Christianne had received over 30 middle ear operations. Between these operations for conductive hearing loss and her use of hearing aids for her sensory neural hearing loss, she began to speak more clearly, especially when singing songs. She also benefitted from glasses for her visually disabling refractive errors. We all discovered that, with her glasses, she did not have to watch Sesame Street with her face very close to the screen, and she more clearly saw the pictures in her board books without bringing the book very close to her face. Although she is considered legally blind, her functional vision did not prevent her from learning from educational television or learning from board books. These functional outcomes with assistive technologies were critical to her ongoing learning. These experiences also emphasize that physicians should monitor not only growth and nutrition but also vision and hearing and the potential of common comorbidities (e.g., seizures, sleep disorders) to slow developmental progress.

***Lesson 5: Preschool Education Promotes Learning Developmental Skills***

When Christianne was born, there were no early intervention programs, head start centers for children with disabilities, or special education services via PL 94–142 [8,44,45]. For early intervention services, she went to a “Center for Dysfunctional Children”. This program had been established by the association of retarded children (now ARC) and offered parent mediated interventions for play, communication, and adaptive skills [41,42]. It also gave my parents and siblings activities that we could do at home. These supportive experiences provided a road map of using play and daily activities for learning.

When Christianne turned 4 years old, my mother enrolled her in the local Montessori with younger typical preschool peers. Outside of preschool, Christianne received speech therapy and audiological interventions. She loved to ride her big wheel tricycle to the local playground. We discovered that these community activities allowed Christianne opportunities for motor and social learning. Some of the multidimensional components of her health, activities, and participation during key developmental periods are illustrated by applying the International Classification of Functioning, Disability and Health for Children and Youth (ICF-CY) model [1,36,46].

**Figure 1:**
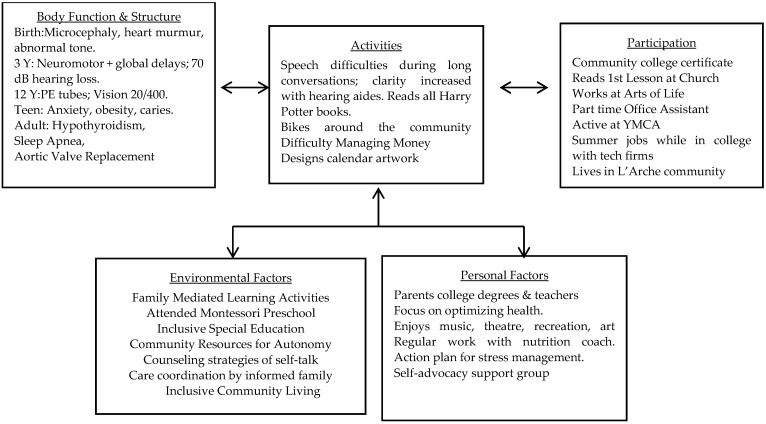
ICF Lifecourse model for Christianne through age 54, years after late prematurity.

In this model, the components of health include body functions and body structures; activity and activity limitations; participation and participation restrictions; and environmental factors. The environment is formed by the physical, social, and attitudinal environments in which children, adolescents, and young adults live and conduct their lives. The ICF framework goes behind the dichotomous classification of impairments (e.g., Intellectual Disability, developmental coordination disorder, legal blindness, 70 db hearing loss) and instead describes a spectrum of functioning at body structure and body function levels (impairment impact on organ systems, brain, or psychological functioning). Activities are whole-person tasks like running, reading, communicating, and dancing. Participation includes roles with peers like being on a team, participating in religious activities, or meeting friends for a movie or dinner. The model illustrated in Figure 1 has been applied to Christianne’s trajectories across her lifecourse and include her complex medical and developmental sequelae after a late preterm birth in a setting of neurodiversity.

Using a Life Course Health Development framework to analyze the origins and impact of prematurity provides opportunities to optimize health and the spectrum of developmental, behavioral, and social outcomes. These opportunities are critical for increasing resilience, addressing social determinants of health, and promoting equity after neonatal critical illness.

## 6. Middle Childhood: Peers and Siblings Are Essential Role Models

With PL 94–147, she was able to attend inclusive special education in her community with 6-year-old peers [44]. By age 8, Christianne could read sight words, perform basic math operations in addition and subtraction, and spell words accurately. She attended art and music lessons, as did her sisters. During this time, she experienced the value of community peers. The impact of these friends is best illustrated by how she learned to ride a two-wheel bike. At age 4, Christianne competently rode a big wheel bike on the sidewalk that circled the *cul-de-sac* on our street and served as the neighborhood bike track. She could drive at a good speed and kept up with older siblings. Over time, all her sisters tried very hard, without success, to persuade Christianne to learn to ride a two-wheel bike like them. This occurred over several summers. Then, one summer day, Shirley, her neighbor friend said, “Christianne, only younger kids use big wheels; you are ready for trying a 2-wheel bike.” Within 24 hours, Christianne began to use a 2-wheel bike with training wheels. With practice, she set a goal of becoming free of training wheels. Within a month, she could ride a 2-wheel bike independently. Thus, we all learned that Christianne could learn from community experiences and from the motivation of keeping up with her peers [47].

***Lesson 6: Activity promotes fitness, fun, and community participation.***

As part of Christianne’s challenges with developmental disabilities, she experiences balance and coordination difficulties. In current parlance, she was a premature infant with a developmental coordination disorder. She was also a premature infant with global developmental delays. But what did these terms mean? By going to playgrounds, swimming, and learning to ride a bike, Christianne’s skills became more functional. By learning to play the piano and taking art classes, her fine motor coordination skills improved. Life was not therapy without success but regular activities that were fun and had an impact on her health and well-being. The most important lesson from learning to ride a bike was the increased community participation that followed. All of Christianne’s siblings went swimming at the community pool and rode their bikes one mile to arrive there. All of them went to the local library half a mile away. Now, Christianne was able to do both of these activities. In the process, her siblings coached her to cross streets carefully, ride in bike lanes, lock her bike, and more. She was able to learn to become independent in the community in a safe and mentored manner.

## 7. Adolescence: Health, Behavioral and Community Supports

Because she learned to swim, Christianne participated in the Special Olympics and won numerous medals. She learned to read music and had a favorite Bach song she was proud to play on the piano. She participated in community recitals. However, she had a tendency to be rebellious and struggled with keeping up with peers. She needed an approach to maximize her academics, build her self-confidence and self-direction, and optimize her academic practical learning. She left her local school district to attend a comprehensive skill-building program for learning and independence. The program was run by Dominican Nuns who emphasized neatness, participation in chores, forming friendships, negotiating relationships, learning to use the keyboard, and completing projects. With this scaffolding, she returned to her local high school and graduated at age 21.

During adolescence, there was ongoing emphasis on practical applied academics and being prepared for independent community living. In addition, ongoing social skills expanded as Christianne enjoyed participating in plays, joined 4-H, and attended summer camp.

**Adolescent health trajectories**. Her vision meets the criteria of legal blindness because of retinopathy and high myopia. Her aortic valve dysfunction was monitored with echocardiography. She needed supports for managing dysmenorrhea. She needed coaching for wise nutrition, as she binged on junk food. She required extensive dental surgery for caries and orthodontia for malocclusion. These medical needs were not that different from most teens. However, during health encounters with subspecialists, she benefitted from my parents’ meticulous record-keeping and their informing of physicians about changes in her typical performance. Most importantly, she needed counseling strategies for managing anxiety and benefitted from an adolescent support group.

***Lesson 7: One will not remain a child at home with parents but become an adult with community supports.***

At age 20, her 19-year-old sister left for college. For the first time, Christianne was home alone. She continued to need behavioral interventions for anxiety. She needed coaching for executive dysfunction. She underwent a comprehensive neuropsychological assessment. The results indicated an IQ within the mild intellectual disability classification, with challenges in working memory and language processing. Her academic skills included reading, 6th grade; mathematics, 3rd grade; and written language, 8th grade. In adaptive skills, her Self-Care Standard Score was 80. Her scores for communication were 70, social—72, and community living—68. The mean(sd) for these scores was 100(15) [48]. This profile is a far cry from her initial prognoses of severe intellectual disability in the neonatal intensive care nursery An important lesson for health professionals is to understand that these numbers do not really indicate what Christianne can do consistently and independently. She has read all the Harry Potter books. She reads regularly during the first lesson at church on Sunday. She has a network of friends that enjoy dining out, going to movies, bowling, and taking evening swims. She can take public transportation across the city and volunteers in a day care as an assistant teacher. Not one cognitive, adaptive, or academic achievement score predicted the activities Christianne can perform in the community. The message for health professionals is also clear. These interdisciplinary assessments are opportunities to build on current strengths and, in areas where there have been challenges, devise a comprehensive plan to promote learning and independence [49,50,51].

***Lesson 8: Develop a plan for life after high school.***

Christianne arrived at the crossroads of leaving high school. She articulated four adult goals:

1. To attend community college.
2. To learn job skills that would allow for paid employment.
3. To continue to participate in music recitals, theatre productions, and social activities with friends.
4. To become more active in managing her health with respect to nutrition, sleep, and the management of stress.

After careful searching, she attended a community college program that was accessible and utilized coaching and mentoring. Christianne has always lacked a sense of satiety, and, without structure and supports to limit her access to junk food, she would consume all available food. Christianne’s program involved living in a dorm with residential assistants. She had full access to an open cafeteria. This access effectively ended all control over her late-night binge eating. In examining this issue, it would have made an enormous difference to have understood the impossibility of her developing the self-regulation of appetite and to have had more effective professional advice about behavioral interventions at a much earlier age. The lesson for health professionals is to not passively accept severe obesity as inevitable.

After two years, Christianne received an associate degree certificate. Her vocational skills included being a hospital transport orderly, clerical skills for being an office assistant, and skills in being a preschool teacher’s aide. She participated in self-advocacy for citizens with intellectual disabilities. She began to reside in a L’Arche inclusive community of caring for individuals with and without intellectual disabilities who self-managed their community home, worked through supportive employment, and participated in the community activities of dining, recreating, and worshipping.

## 8. Adult Health and Social Challenges

***Lesson 9: Physical and behavioral health needs do not end at age 21.***

A variety of medical and behavioral health disorders were identified while in college. She developed autoimmune thyroiditis and began to undergo thyroid replacement therapy. She continued to benefit from counseling and behavior health group sessions. She received counseling in motivation interviewing and cognitive behavior therapy. She began to take an SSRI medication for anxiety. She learned to self-talk to manage her anxiety. Her behavior management plan provided positive support to decrease stressors and increase group opportunities for cooperative peer relationships. She learned to be accountable for household chores, regularly contributed to meal preparation, and had specific responsibilities during grocery shopping. She attended church regularly and participated in YMCA and community recreational activities. In these experiences, she needed both mentoring and informed supervision.

In 2001, my father died at age 75 after a long illness. Christianne was 32 years old. She mourned. She missed her daily walks of 5 miles with her father. After these walks, they would have a shared lunch. She wept often about missing these walks and father–daughter conversations. She experienced difficulties with sleep. She kept on asking housemates and family members to visit the cemetery to pray and say her goodbyes. She always told others she met how much she missed her father. She began to binge eat an enormous quantity of evening food. Over the course of one month, she gained 20 pounds. By 2 months, her weight gain was up 40 pounds. A psychology evaluation revealed anxiety and depression. As she had disrupted sleep and had begun to snore, a polysomnogram revealed significant sleep apnea. She learned to use a positive airway pressure device at night. Her SSRI medication was titrated. Regular walks were resumed. At Arts of Life, she drew pictures of heaven and angels.

With these supports, she began to heal. The lesson for health professionals is not to underestimate loss and depression and to consider its hidden presence in individuals with developmental disabilities. Like their neurotypical peers, this can be summarized as

***Lesson 10: Young adults require proactive integrated physical and behavioral health services.***

**Adult health challenges**. At age 48, Christianne experienced cardiac syncope during exercise and was diagnosed with critical aortic insufficiency. She required open-heart aortic valve replacement. Prior to her operation, she met with the cardiac surgeon to discuss the risks and benefits of the operation. He told her that her aortic valve needed replacement. She asked what needed to be done to fix it. He said he would perform an operation, she would not feel pain, she would have a scar on her chest, and she would get a new valve. He asked if she had any questions. Christianne said she had two questions. First, how many times had the surgeon done this operation? The surgeon holding her hand at eye level told her he had done this operation over 1000 times. She said “that is good, you know what to do. “The second question was personal, she said. The surgeon said “do not worry; I am your doctor; you can tell me about personal things.” Christianne said she knew she would be asleep during the operation but wondered what would happen if she needed to use the bathroom. She did not want to embarrass herself by having an accident with bowel or bladder control. The surgeon said “do not worry; during heart surgery, we make sure we collect your urine; you will not have #1 accidents. As for pooping, you must be without food for 12 h, and the anesthesia slows down your colon. You will not have a poop accident. “She thanked him for his explanations. He asked if there was anything else. She said “where do I and my brother sign?” We both signed the consent.

Christianne’s open-heart operation required less than an hour of bypass. She was extubated in the recovery room without complications. I had instructed the nursing staff that, without glasses, she was legally blind, and, without hearing aids, she could not hear ordinary verbal requests. Most importantly, she did not do well with surprises. I informed the nursing staff to draw close to her, to use light touch when explaining things to her, and to hold her hand when there was a procedure that caused pain. With this approach and laminated bedside cards to insure consistency across nursing shifts, she was able to understand and cooperate with any phlebotomies, injections, or uncomfortable hospital procedures.

At 48 h post operation, all invasive central lines and tubes were removed. She went to the bathroom on her own. The staff said she was ready to advance her diet. The dietary staff offered Jello, apple sauce, and beef broth. She said apple sauce was for babies. She would try the Jello because it was easy to swallow. She disagreed about the beef broth. “I need real protein to heal, Christianne said.” “How about a roast beef sandwich?” The nurses laughed and agreed with her wisdom.

Within 96 h, Christianne was ready to return home. However, because of her sternotomy, she would require 2 h of personal assistance and some home-visiting nursing care. The state claimed it would take 4 weeks to find anyone for these community supports. A family huddle occurred. There was an occupational therapist attending graduate school in our community. She liked the idea of moonlighting at $25 per hour for 2 h per day. The cost for 10 days was $500. My parents’ trust fund covered it. This saved the state several thousands of dollars in extra costs for awaiting discharge. It also helped my sister on her road to recovery. This event also highlights the drastic limitations in essential community services for individuals with and without disabilities [30].

**Figure 2:**
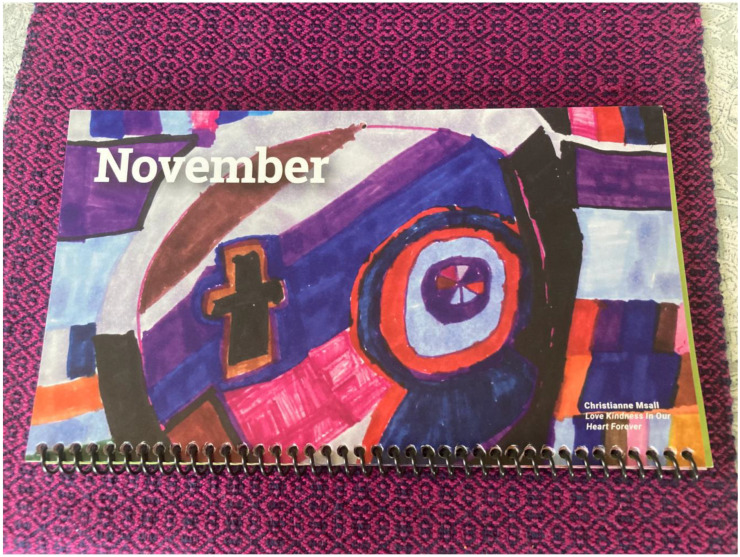
The artwork of Christianne used for an Arts of Life (Calendar).

Postoperatively, she went to a cardiac rehabilitation program. Christianne lost weight, implementing a community-wide safety plan for limiting access to food by insisting on no meals without a companion. Her sister Karin coordinated all her follow-up medical and rehabilitation care. There was no involvement of any care coordination from the current health system. She resumed part-time office clerical work and part-time work at Arts of Life. Figure 2 is the picture she drew at Arts of Life after its reopening after the COVID-19 lockdown. Christianne’s narrative for this picture was, “When I look at my work, I see how happy I am, my positivity. When others look, they see how wonderful I am, that I have good ideas and techniques. I am proud very proud of myself”.

## 9. Impact on Family

My brother, sisters, and I have learned the value of lived experiences in joining Christianne on her lifecourse journey. We have experienced the value of accessible community support for Christianne’s participation in community life [51]. In health care, we have found that adults with developmental disabilities experience diagnostic overshadowing and limited access to subspecialty care, behavioral health, and care coordination. Christianne requires a health system that is informed about exceptional needs and can advocate for solutions to the discrimination of long waiting lists for community care and personal care assistance.

## 10. Summary

My family and I have been on a 54-year journey. I would suggest that there are 9 lessons that my sisters, brother, and I have learned.

1. Parent and sibling support matters.
2. All children, teens, and adults continue to learn.
3. The community journey has many bumps in the road, but these potholes are opportunities for recalibrating supports for success.
4. Remember that optimal health is not achieved by focusing on deficit models in medicine, education, or vocational training.
5. The key framework based on ICF principals is to manage impairments that impact health (vision, hearing, hypothyroidism, critical aortic stenosis), optimize functional strengths for independence, and promote social learning experiences for participation (L’Arche Supported Living, Arts of Life, church, theaters, support groups). These strategies are highlighted in the ICF Model of Figure 1 and emphasize that support resources need to be continually targeted and accessed over one’s lifecourse [1,36,37,46].
6. Coordinated and compassionate care continues to be required in adulthood. Christianne’s complex care coordination included cardiology, ophthalmology, otolaryngology, audiology, dentistry, endocrinology, internal medicine, and psychiatry.
7. Christianne requires health care professionals who engage her, understand her strengths and challenges, and become updated by the ongoing involvement of family and community support, who know her as a person.
8. The key is to communicate in a respectful and shared decision-making manner respecting a life of neurodiversity [11,12,13].
9. Beware of diagnostic overshadowing. Christianne is more than her genetic diagnoses, prematurity, or IQ score.

## 11. Conclusions

These shared voyages have allowed me to better understand lived experiences about the facilitators and barriers for optimizing Christianne’s physical, developmental, behavioral, and social functioning. My advice to health professionals is as follows: until you find out how the child and family develop over time, be careful about prognosticating the future. In addition, make efforts to follow up on these children after they leave the hospital, similar to how children and adults with life-threatening malignancies find out what has happened and how the family is doing five years later and beyond. These touchpoints are opportunities to engage families, link them to community support, and learn about journeys of neurodiversity and exceptionality. This will render you better physicians and demonstrate your caring. Failure to remain both skilled and caring will have negative impacts on children and families. You will too often leave families isolated and without support. Remember, all parents need support and access to ongoing care, which includes health, developmental, psychological, and community supports. Be humble; you can be uncertain, but also know you have a critical role for hope and for learning with families about the complexity of developmental trajectories. Remember that isolation and hopelessness do not lead to positive outcomes for either children who are critically ill or their parents and brothers and sisters. In an era of complex time demands and fragmented systems of care, continue to care by touching base after discharge. In partnerships with families, your obligation is not to fix every risk but to promote both support and community handoffs, so that you optimize functioning, participation, and family well-being. Never forget that health develops as an active process across the entirety of the lifecourse, and that, regardless of diagnosis, much can be done at every stage of life to help optimize physical, mental, social, emotional, and spiritual health. Through Christianne, I have discovered the value of hope, quality medical care, and behavioral support. Optimizing health is our task as health professionals, and this helps us remember that medicine requires a journey of caring and advocacy.

## Figures and Tables

**Table 1:** Enablement framework for communicating about lifecourse neurodiversity after prematurity.

1	Children with disabilities are humans that are capable.
2	Educate yourself about community resources beyond the hospital.
3	Beware of predicting what children will specifically do in their developmental and functional skills in the future.
4	Debrief about causal pathways: Rarely does one specific medical event determine all.
5	Disability is a lived experience.
6	Emotions, kindness, hope, and relationships over time matter.
7	One’s role is not to be an all-knowing physician or perfect parent.
8	Beware of simplistic answers for parental coping not grounded in accessible resources.
9	Emphasize parental self-care, balance, and well-being.
10	Expect anger, overwhelmedness, depression, and anxiety.
11	These touchpoints are opportunities for care and support.
12	Focus on what is achievable; prioritize successes that provide hope.

The framework for these principles is based on references [3,11,12,19,20,25,26] as well as the lived experience of the author.

## Data Availability

The data presented in this study are available in article. Christianne’s Artwork for the 2024 Arts of life calendar can be viewed at the following link: Arts of Life <info@artsoflife.org>. The 2023 annual report of Arts of Life a 504C Non for Profit can be obtained at https://aol-wpe-largefs.s3.amazonaws.com/artsoflife/wp-content/uploads/2023/10/AOL-AnnualReport-Digital-231018-1.pdf?mc_cid=29d1515854&mc_eid=214107a8bc.
